# Downstream Complications and Healthcare Expenditure after Invasive Procedures for Lung Lesions in Taiwan

**DOI:** 10.3390/ijerph18084040

**Published:** 2021-04-12

**Authors:** Szu-Chun Yang, Ching-Han Lai, Chin-Wei Kuo, Chien-Chung Lin, Wu-Wei Lai, Jung-Der Wang

**Affiliations:** 1 Department of Internal Medicine, National Cheng Kung University Hospital, College of Medicine, National Cheng Kung University, Tainan 704, Taiwan; yangszuchun@gmail.com (S.-C.Y.); northhermes@gmail.com (C.-H.L.); kbh557@gmail.com (C.-W.K.); joshcclin@gmail.com (C.-C.L.); 2 Institute of Clinical Medicine, College of Medicine, National Cheng Kung University, Tainan 701, Taiwan; 3 Department of Surgery, National Cheng Kung University Hospital, College of Medicine, National Cheng Kung University, Tainan 704, Taiwan; wwlai@mail.ncku.edu.tw; 4 Department of Public Health, College of Medicine, National Cheng Kung University, Tainan 701, Taiwan

**Keywords:** complication, cost, lung cancer screening, low-dose computed tomography, harm

## Abstract

This study aimed to estimate the downstream complications and healthcare expenditure after invasive procedures for lung lesions, which in turn could be used for future cost-effectiveness analyses of lung cancer screening in Taiwan. We interlinked the Taiwan National Beneficiary Registry with the National Health Insurance Reimbursement databases to identify non-lung cancer individuals aged 50–80 years who underwent invasive lung procedures within one month after non-contrast chest computed tomography between 2014 and 2016. We directly matched one individual with 10 controls by age, gender, calendar year, residence area, comorbidities, and the past one-year healthcare expenditure to calculate incremental one-month complication rates and attributable costs. A total of 5805 individuals who underwent invasive lung procedures were identified and matched with 58,050 controls. The incremental one-month complication rates were 13.4% (95% CI: 10.9% to 15.8%), 10.7% (95% CI: 9.2% to 12.1%), and 4.4% (95% CI: 2.0% to 6.7%) for thoracic surgery, bronchoscopy, and needle biopsy, respectively. The incremental one-month healthcare expenditure for minor, intermediate, and major complications were NT$1493 (95% CI: NT$-3107 to NT$6092), NT$18,422 (95% CI: NT$13,755 to NT$23,089), and NT$58,021 (95% CI: NT$46,114 to NT$69,929), respectively. Individuals aged 60–64 years incurred the highest incremental costs. Downstream complications and the healthcare expenditure after invasive procedures for lung lesions would be substantial for non-lung cancer individuals 50–80 years of age. These estimates could be used in modeling the cost-effectiveness of the national lung screening program in Taiwan.

## 1. Introduction

Low-dose computed tomography (LDCT) screening has been shown to reduce lung cancer mortality among high-risk populations [1,2]. The evolving evidence, guidelines, and policies have increased the use of chest computed tomography (CT) for lung cancer screening [3,4]. One of the drawbacks of lung cancer screening is its high rate of false-positive results [1,5], which brings about unnecessary invasive lung procedures, including thoracic surgery, bronchoscopy, and needle biopsy. In Taiwan, for example, a 6.1-fold increase of CT-guided needle biopsies was noted within 10 years [6]. As a result, an increase in complications has been observed, resulting in additional harm and increasing the healthcare expenditure.

The Taiwan National Lung Screening Program among high-risk participants who have never smoked began in 2014 and ended its enrollment in 2019 [7], with the results being disclosed in the coming years. However, before crafting the lung cancer screening program into policy, its cost-effectiveness needs to be estimated. To study the cost-effectiveness, downstream complications and healthcare expenditure after invasive procedures for false-positive lung lesions need to be explored. According to the U.S. experience, the complication rates from invasive diagnostic procedures were 8.5–9.8% in the National Lung Screening Trial (NLST) [5]. Other researchers, using data from a community setting, have estimated the rates to be 22.2–23.8% and the associated costs to range from US$6320 for minor complications to US$56,845 for major complications [8]. However, values based on the U.S. population might not be applicable to the LDCT screening-eligible population in Taiwan. Moreover, the community-based study did not restrict the invasive procedures to those performed immediately after non-contrast chest CT. Additionally, complications usually occur shortly after invasive procedures, a one-year observation period the study used to identify the events seemed longer than our understanding.

We hypothesized that downstream complications and the healthcare expenditure after invasive procedures for false-positive lung lesions in Taiwan would be substantial. Based on the incremental approach presented by Huo et al. [8] and Taiwanese nationwide interlinked data, we tried to estimate the one-month complication rates and costs after invasive lung procedures, which could be used for future cost-effectiveness analysis of lung cancer screening in Taiwan.

## 2. Methods

### 2.1. Data Source

We interlinked the Taiwan 2014–2016 National Beneficiary Registry database with the 2013–2017 National Health Insurance (NHI) Reimbursement database to identify the study and 1:10 matched cohorts for incremental comparison. These databases capture the claims data of 99% of the nation’s inhabitants and are representative of the Taiwanese population [9]. The Institutional Review Board of National Cheng Kung University Hospital approved the study before its commencement (B-EX-109-038). Informed consent was waived because of the use of de-identified information.

### 2.2. Study Cohort Undergoing Invasive Procedures after Non-Contrast Chest CT

Since the Taiwan National Lung Screening Program allowed participants with a family history of lung cancer to be 50–75 years of age [7], and since the guidelines recommended that adults can receive LDCT screening up to the age of 80 [10], individuals aged 50–80 years who underwent invasive lung procedures during 2014–2016 were identified. We adopted the categorization of the NLST [1] and stratified invasive lung procedures into three groups: Thoracic surgery, bronchoscopy, and needle biopsy. Two pulmonologists mapped the procedures to claim codes reimbursed in Taiwan’s NHI (see Supplementary Table S1). For those who received more than one kind of procedure, the most invasive one was selected. We defined thoracic surgery as being more invasive than bronchoscopy, and bronchoscopy as more invasive than needle biopsy. The first-encountered date of the selected procedure was denoted as the index date of each individual. Individuals must undergo non-contrast chest CT (NHI claim code of 33070B) within one month prior to the procedure, and those with claims of intensive care within one month prior to the index date were excluded. By linking the data to the 2013–2017 National Cancer Registry database, we limited individuals with no diagnosis of lung cancer 12 months before and after the index date. To ensure the same follow-up period and unbiased results of the complications rates, we also interlinked the data to the National Mortality Registry database and limited individuals to those who survived for more than one month after the index dates. The study group individuals’ demographic characteristics, including calendar year of the procedure, age, and gender, were assessed. To adjust for socioeconomic class, residence was categorized into urban, sub-urban, and rural areas according to previous researchers’ classifications [11]. Comorbidities were retrieved from inpatient and outpatient claims within one year prior to the index date and scored according to the Charlson Comorbidity Index [12,13]. The healthcare expenditure one year prior to the index date was also calculated. Smoking status was not analyzed for lack of such data.

### 2.3. Matched Cohort Randomly Selected from the General Population

Since post-procedural complications and healthcare expenditure might not necessarily be attributable to the invasive lung procedure itself, we applied an incremental approach to subtract the effects unrelated to the procedure. That is, we matched every index subject to a fixed ratio of comparable controls who did not undergo these procedures. The differences in downstream complications and healthcare expenditure between the study and matched cohorts were attributed to the invasive lung procedure. The incremental approach has been widely adopted in health services research [8,14]. The date on which the procedure was claimed was the index date for the study individual, whereas 1 January in the year of the index procedure was assigned as the pseudo-index date for those who did not undergo a procedure. We matched one study individual with 10 controls randomly selected from the general population, who also had no diagnosis of lung cancer 12 months before and after the pseudo-index date, and survived more than one month after the pseudo-index date, without replacement. The selected covariates for matching included the calendar year, age, gender, residence area, Charlson Comorbidity Index, and past one-year healthcare expenditure upon the index or pseudo-index date.

### 2.4. Downstream Complications and Healthcare Expenditure

A total of 43 kinds of complications, classified as major, intermediate, or minor, were reported in the NLST [1]. Previous investigators mapped the complications to the *International Classification of Diseases, Ninth Revision* (*ICD-9*) codes [8]. We further mapped the *ICD-9* diagnosis and procedure codes to the *ICD-10* CM (clinical modification) and PCS (procedure coding system) codes, as well as the procedure codes of Taiwan’s NHI (see Supplementary Excel for details). Downstream complications refer to the complications claimed after the index or pseudo-index date. If a complication diagnosis was claimed within one month before the invasive lung procedure, it could be the trigger of a procedure, so we did not consider it as a downstream complication. Complication rates were calculated as the number of individuals with a complication within one month divided by the total number of individuals in that category. We subtracted the complication rate of the matched cohort from that of the study cohort to derive the incremental one-month complication rate.

We analyzed the healthcare expenditure rendered on complications and summed up all payments to obtain the one-month downstream healthcare expenditure. Likewise, we subtracted the healthcare expenditure of the matched cohort from that of the study cohort for the incremental one-month costs attributable to complications. All payments in different calendar years were adjusted for medical inflation rates up to the end of 2017.

### 2.5. Statistical Analysis

We did not show the standardized differences between the study and matched cohorts because they were completely matched and the values were 0. We expressed the incremental one-month complication rates and attributable costs as means and 95% confidence intervals (CIs). We used SAS software (version 9.4, SAS Institute, Cary, NC, USA) to perform the analyses.

## 3. Results

### 3.1. Study and Matched Cohorts

A total of 7,880,647 beneficiaries aged 50–80 years in 2014–2016 were identified. Among them, 31,217 individuals underwent at least one invasive lung procedure, and 5805 subjects received the procedures within one month following non-contrast chest CT outside intensive care units. Table 1 shows that the study individuals receiving the procedures were older, more likely to be male, had higher comorbidity scores, and incurred a higher healthcare expenditure in the past one year. In the study cohort, 1263, 3417, and 1125 individuals underwent thoracic surgery, bronchoscopy, and needle biopsy, respectively. The individuals receiving thoracic surgery were much younger, more likely to live in urban areas, and had lower comorbidity scores than those undergoing other invasive lung procedures. The 5805 individuals were 1:10 directly matched by calendar year, age, gender, residence area, comorbidity scores, and past one-year healthcare expenditure to individuals who had not received the procedures. All selected covariates of the study and matched cohorts were completely matched (Table 2).

### 3.2. Incremental Complication Rates

**Figure 1:**
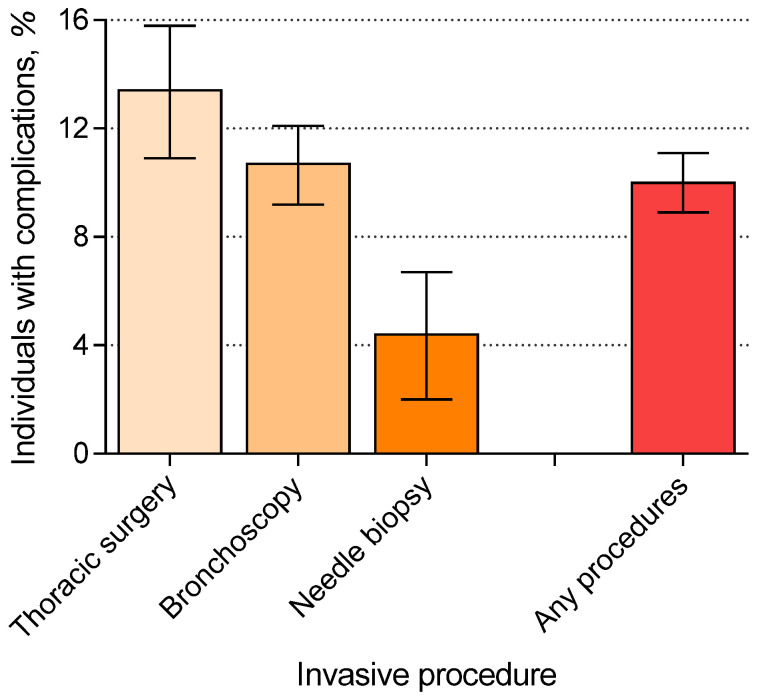
Incremental one-month complication rates (95% confidence intervals) by procedure.

Figure 1 shows the incremental one-month complication rates by procedure. The incremental one-month complication rates were 13.4% (95% CI: 10.9% to 15.8%), 10.7% (95% CI: 9.2% to 12.1%), and 4.4% (95% CI: 2.0% to 6.7%) for thoracic surgery, bronchoscopy, and needle biopsy, respectively. The incremental one-month complication rates stratified by different age groups are detailed in Supplementary Table S2. Although the minor complication rate was higher in individuals receiving thoracic surgery, the intermediate and major complication rates were lower than those undergoing bronchoscopy.

### 3.3. Incremental Costs Attributable To Complications

**Figure 2:**
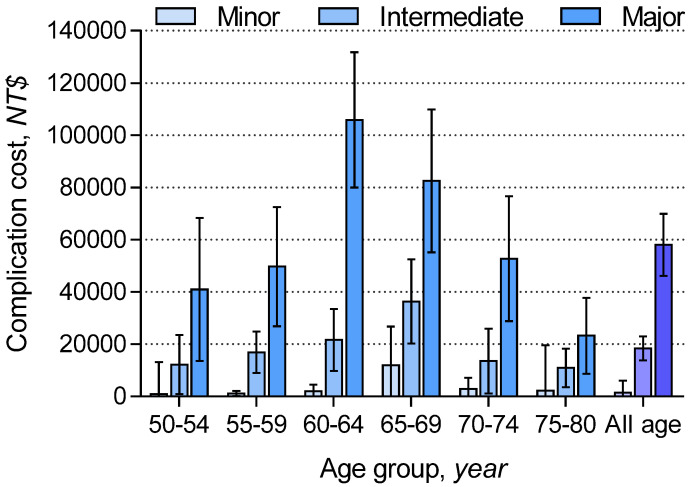
Incremental one-month complication costs (95% confidence intervals) by the severity of complications, stratified by age group. NT$30 = US$1.

Figure 2 illustrates the incremental one-month costs attributable to complications by the severity of complications, stratified by age group. The study group individuals experiencing major complications incurred a higher incremental healthcare expenditure than those with intermediate or minor complications. Individuals aged 60–64 years with complications had the highest incremental costs. The incremental one-month healthcare expenditure costs among all age groups were NT$1493 (95% CI: NT$-3107 to NT$6092), NT$18,422 (95% CI: NT$13,755 to NT$23,089), and NT$58,021 (95% CI: NT$46,114 to NT$69,929) for minor, intermediate, and major complications, respectively. Supplementary Table S2 also details the incremental one-month complication costs stratified by different types of procedures. Individuals receiving thoracic surgery incurred a lower incremental healthcare expenditure than those undergoing bronchoscopy or needle biopsy.

## 4. Discussion

The evolving evidence and guidelines of lung cancer screening have dramatically increased the utilization of chest CT [3,4], which identifies ever more incidental lung lesions and results in an increase in invasive diagnostic procedures. Using the nationwide representative data and an incremental approach to subtract the effects unrelated to invasive lung procedures, this study estimated the rates of downstream complications and the related healthcare expenditure. We found that the percentage of individuals experiencing downstream complications in one month could be up to 10.0% (95% CI: 8.9% to 11.1%; Figure 1), and the one-month healthcare expenditure could be as high as NT$105,856 (95% CI: NT$79,980 to NT$131,731; Figure 2). These values could be used as input parameters in modeling the cost-effectiveness of the national lung screening program in Taiwan.

Consistent with previous reports [8,15], major complications have substantially increased the incremental healthcare expenditure. Huo et al.’s study, using claims data, did not require every individual to undergo non-contrast chest CT within one month before the procedure [8], which in turn might have captured more procedures unrelated to screening. Moreover, the one-year observation period for identifying the complications was too long, which might have resulted in an overestimation of the results. Compared to the incremental costs ranging from US$6320 for minor complications to US$56,845 for major complications [8], our downstream healthcare expenditure of NT$1493 (US$50) for minor complications and NT$58,021 (US$1935) for major complications were much lower, in part because we specified one-month costs.

Individuals aged 60–64 years incurred the highest incremental costs, whereas those older than 70 years of age did not incur greater complication costs than those of the younger age groups (Figure 2). A plausible explanation is that management for the complications of older individuals is more conservative, resulting in a lower healthcare expenditure. Since data relating to performance statuses and pulmonary function tests were not available in the claims data, we were unable to adjust for these variables. Although we excluded invasive procedures performed in intensive care units, individuals receiving thoracic surgery were inherently healthier, and the performance statuses and pulmonary functions were better than those of subjects undergoing other invasive procedures. Moreover, the surgeries were most often performed by experienced doctors in academic institutions. Consequently, the major complication rates and the related healthcare expenditure of individuals receiving thoracic surgery appeared lower than those of the others.

A previous Taiwanese study estimated the rate of major complications after percutaneous lung biopsy to be 0.1% to 1.6% [6], whereas the downstream major complication rate for needle biopsy among all age groups was 1.8% (95% CI: 0.4% to 3.2%) after adjustment for the baseline rate in our study (see Supplementary Table S2). This inconformity might be explained by some differences in study design. The previous study only selected pneumothorax requiring drainage, hemoptysis, cardio-pulmonary resuscitation, and exploratory thoracotomy as major complications, and analyzed all individuals older than 20 years. In contrast, our study used multiple claim codes based on the NLST to define the major complications [8], and limited individuals to those of 50–80 years of age. The results thus seemed acceptable.

We used Taiwan’s NHI reimbursement data to estimate the downstream healthcare expenditure, which captured most of the direct medical costs and included both outpatient and inpatient healthcare services [16]. However, out-of-pocket costs were not included in the estimation. Although one of our previous studies found that out-of-pocket money paid by lung cancer patients was approximately 1/4 to 1/2 of the direct medical costs [17], we believe that the out-of-pocket costs among the non-lung cancer study cohort were relatively small. Our cost estimates could be directly used for cost-effectiveness analyses conducted from a public healthcare sector’s perspective.

Several limitations must be acknowledged in our study. First, although individuals underwent non-contrast chest CT within one month prior to the procedure, and we limited the individuals to those without lung cancer 12 months before and after the index date to mimic participants with false-positive results after lung cancer screening, we might have included individuals who underwent the invasive procedures because of reasons other than a diagnostic work-up for lung lesions. Nevertheless, as we applied the same NHI procedure codes for reimbursements to identify diagnostic or non-diagnostic invasive lung procedures, the downstream complication rates and healthcare expenditure would be similar. On the contrary, our estimates provide healthcare providers and policymakers with insights relating to the potential harms and financial burdens after invasive lung procedures, whether or not they are used for diagnostic or non-diagnostic purposes. Second, although the National Lung Screening Program in Taiwan only enrolled high-risk individuals who had never smoked [7], smoking history was not considered in this study because of the lack of data. This may have resulted in an overestimation of the results, because smoking individuals with related chronic lung disease tend to have a higher rate of procedure-related complications [18,19]. However, the smoking rates for men and women in Taiwan are less than 30% and around 5%, respectively [20], and since our study and matched cohorts were balanced in comorbidities, including chronic obstructive pulmonary disease, the results would not have been overly biased. Third, this study only estimated the complication rates and costs after invasive lung procedures; we did not incorporate effectiveness [7,16] and health utility [21] estimates to determine if LDCT screening is recommended in Taiwan. Future research may take these parameter values into consideration to evaluate the cost-effectiveness. Finally, one must always be cautious when using claims data to draw any causal inferences of downstream complications from the preceding procedures. In this study, however, we applied an incremental approach to adjust for the underlying rates and costs so that the effects unrelated to the procedures would be minimized.

## 5. Conclusions

In conclusion, downstream complications and the healthcare expenditure after invasive procedures for lung lesions would be substantial among beneficiaries 50–80 years of age eligible for LDCT screening. These estimates could be used in modeling the cost-effectiveness of national lung screening program in Taiwan.

## Figures and Tables

**Table 1:** Characteristics of the beneficiaries by procedure within one month after non-contrast chest CT.

	Study Cohort (*n* = 5805)						Without Procedures ^a^ (*n* = 7,849,430)	
	Surgery (*n* = 1263)		Bronchoscopy (*n* = 3417)		Needle Biopsy (*n* = 1125)			
	*n*	*%*	*n*	*%*	*n*	*%*	*n*	*%*
Calendar year								
2014	380	30.1	1072	31.4	357	31.7	7,051,719	89.8
2015	444	35.2	1129	33.0	346	30.8	406,912	5.2
2016	439	34.8	1216	35.6	422	37.5	390,799	5.0
Age, years								
50–54	277	21.9	482	14.1	140	12.4	2,581,833	32.9
55–59	264	20.9	528	15.4	174	15.5	1,688,033	21.5
60–64	257	20.4	625	18.3	191	17.0	1,423,362	18.1
65–69	200	15.8	518	15.2	212	18.8	850,932	10.8
70–74	143	11.3	584	17.1	207	18.4	684,706	8.7
75–80	122	9.7	680	19.9	201	17.9	620,564	7.9
Gender								
Male	791	62.6	2059	60.3	665	59.1	3,787,622	48.3
Female	472	37.4	1358	39.7	460	40.9	4,061,808	51.8
Residence								
Urban	778	61.6	1998	58.5	669	59.5	4,825,156	61.5
Sub-urban	370	29.3	961	28.1	310	27.5	2,293,026	29.2
Rural	115	9.1	458	13.4	146	13.0	731,239	9.3
Unknown	0	0	0	0	0	0	9	0.0
CCI score								
0	405	32.1	928	27.2	252	22.4	5,100,874	65.0
1	295	23.3	828	24.2	208	18.5	1,472,067	18.8
2	202	16.0	596	17.4	204	18.1	683,080	8.7
≥3	361	28.6	1065	31.2	461	41.0	593,409	7.6
Past one-year healthcare expenditure ^b^								
1st quintile	89	7.0	189	5.5	43	3.8	1,584,424	20.2
2nd quintile	92	7.3	222	6.5	66	5.9	1,583,674	20.2
3rd quintile	145	11.5	401	11.7	88	7.8	1,581,260	20.1
4th quintile	269	21.3	683	20.0	188	16.7	1,575,448	20.1
5th quintile	668	52.9	1922	56.3	740	65.8	1,524,624	19.4

^a^ Pseudo-index dates were not assigned before matching; the calendar year, age, residence, CCI score, and past one-year healthcare expenditure were based on the first month that appeared in the 2014–2016 National Beneficiary Registry database. ^b^ 1st quintile: NT$0–2816; 2nd quintile: NT$2817–8504; 3rd quintile: NT$8505–17,062; 4th quintile: NT$17,063–35,084; 5th quintile: ≥NT$35,085. CCI, Charlson Comorbidity Index; CT, computed tomography.

**Table 2:** Characteristics of the study and 1:10 matched cohorts.

	Study Cohort (*n* = 5805)		Matched Cohort (*n* = 58,050)	
	*n*	*%*	*n*	*%*
Calendar year				
2014	1809	31.2	18,090	31.2
2015	1919	33.1	19,190	33.1
2016	2077	35.8	20,770	35.8
Age, years				
50–54	899	15.5	8990	15.5
55–59	966	16.6	9660	16.6
60–64	1073	18.5	10,730	18.5
65–69	930	16.0	9300	16.0
70–74	934	16.1	9340	16.1
75–80	1003	17.3	10,030	17.3
Gender				
Male	3515	60.6	35,150	60.6
Female	2290	39.4	22,900	39.4
Residence				
Urban	3445	59.3	34,450	59.3
Sub-urban	1641	28.3	16,410	28.3
Rural	719	12.4	7190	12.4
CCI score				
0	1585	27.3	15,850	27.3
1	1331	22.9	13,310	22.9
2	1002	17.3	10,020	17.3
≥3	1887	32.5	18,870	32.5
Past one-year healthcare expenditure ^a^				
1st quintile	321	5.5	3210	5.5
2nd quintile	380	6.5	3800	6.5
3rd quintile	634	10.9	6340	10.9
4th quintile	1140	19.6	11,400	19.6
5th quintile	3330	57.4	33,300	57.4

^a^ 1st quintile: NT$0–2816; 2nd quintile: NT$2817–8504; 3rd quintile: NT$8505–17,062; 4th quintile: NT$17,063–35,084; 5th quintile: ≥ NT$35,085. CCI, Charlson Comorbidity Index.

## Data Availability

The datasets generated and/or analyzed during this study are not publicly available due to confidentiality reasons, but the sufficiently aggregated data used for analyses may be available from the corresponding author upon reasonable request.

## Supplementary Materials

The following are available online at https://www.mdpi.com/article/10.3390/ijerph18084040/s1, Table S1: Procedure codes of invasive lung procedures; Table S2: Incremental one-month complication rates and attributable costs by procedure and age group; Supplementary Excel: *Mapping International Classification of Diseases*, *Ninth Revision* (ICD-9) codes to *ICD-10* and National Health Insurance (NHI) codes for complications.
